# Antibody–Drug Conjugates: Pharmacotherapeutic Properties and Future Perspectives

**DOI:** 10.3390/pharmaceutics18040468

**Published:** 2026-04-12

**Authors:** André Augusto, Maria L. S. Cristiano, Jaime Conceição

**Affiliations:** 1 Faculty of Sciences and Technology, Universidade do Algarve, 8005-139 Faro, Portugal; a71282@ualg.pt (A.A.); mcristi@ualg.pt (M.L.S.C.); 2 Centre for Marine Sciences (CCMAR), Universidade do Algarve, 8005-139 Faro, Portugal; 3 Algarve Biomedical Centre Research Institute (ABC-Ri), Universidade do Algarve, 8005-139 Faro, Portugal; 4 Centre for Interdisciplinary Studies (CEIS20), Universidade de Coimbra, 3000-457 Coimbra, Portugal

**Keywords:** antibody–drug conjugates, future perspectives, mechanisms of action, pharmacotherapy, pharmacovigilance

## Abstract

**Background:** The clinical landscape for antibody–drug conjugates (ADCs) is currently experiencing an unprecedented expansion, with more than 20 agents approved to date and hundreds presently under clinical evaluation, underscoring their growing impact in precision oncology. By combining the cytotoxic potency of chemotherapy with the selectivity of monoclonal antibodies, ADCs have redefined targeted cancer therapy. Nevertheless, challenges related to toxicity, resistance, and suboptimal drug delivery continue to limit their full clinical potential. **Objectives:** This review provides a comprehensive description of currently approved ADCs, with a particular focus on their pharmacotherapeutic properties, mechanisms of action, therapeutic indications, and safety profiles. By integrating currently available clinical data and pharmacological properties, it is possible to identify key translational gaps between ADC design and their real-world performance. This article also evaluates how the structural components contribute to both efficacy and toxicity of ADCs, offering a framework for rational molecular optimizations. **Conclusions:** Beyond the current oncology-centric paradigm, this review highlights the imminent pivot toward non-oncology applications, including targeted therapies for autoimmune, infectious, and neurodegenerative diseases. Importantly, this article highlights emerging innovations shaping the next generation of ADCs, including bispecific antibodies, novel cytotoxic payloads with improved therapeutic indices, and advanced linker technologies enabling more precise payload release. Despite current limitations, ongoing advances in ADC development, along with a rapidly expanding clinical pipeline, position these drugs in a dynamic therapeutic class with the potential to transform multiple complex diseases and improve the quality of life of patients who have them.

## 1. Introduction

Cancer remains one of the most devastating diseases worldwide, not only because of its high mortality and morbidity, but also due to the emotional and financial burdens it places on patients, families, and healthcare systems. Although major advances have been made in cancer research, current treatments are mostly ineffective, leading to poor prognosis and disease management. In addition, they are frequently associated with significant undesirable effects that further reduce the quality of life of patients and entail high costs due to drug pricing and the need for repeated administrations [1,2]. Over the years, new cancer therapies have been developed to address the limitations of existing treatments. For example, targeted therapies with antibody–drug conjugates (ADCs) emerged as an innovative way of delivering highly cytotoxic agents, also known as payloads, by taking advantage of the selectivity of certain antibodies to specific receptors that are overly expressed on the surface of cancer cells when compared to their healthy counterparts [3,4]. Although this approach to targeted therapies was first conceptualized in the 60s, based on the “magic bullet” concept of Paul Ehrlich, it was only during the year 2000 that the first ADC (gemtuzumab ozogamicin) obtained its market approval for clinical use [5]. Structurally, ADCs are composed of three main components [6]: (i) an antibody moiety responsible for directing the ADC to the targeted cells, promoting the internalization of the ADC upon interacting with its complementary cell surface receptor or, if not internalized, stimulating the activation of the immune system via antibody-dependent cell-mediated cytotoxicity (ADCC), antibody-dependent cellular phagocytosis (ADCP), and complement-dependent cytotoxicity (CDC); (ii) a certain number of payloads (commonly between two to eight) that induce cell death through their specific mechanisms of action (e.g., they can act as mitotic inhibitors, topoisomerase I inhibitors, or damaging the genetic material of cancer cells); and (iii) a certain number of linkers (equal to the number of payloads) responsible for maintaining the payloads covalently bound to the antibody moiety and for promoting their rapid release whenever the original ADC is exposed to certain chemical or enzymatic stimuli that are frequently found within tumoral microenvironments or inside cancer cells.

The concept of using ADCs to treat some types of cancer seems very promising, as patients can be submitted to highly effective treatments, like the ones used in conventional chemotherapy, while minimizing the risk of adverse side effects, since ADCs use antibody moieties that are very selective to a tumor-associated antigens (TAA), like the ones used in immunotherapies with monoclonal antibodies (mAbs) or, ideally, to a tumor-specific antigen (TSA) [7]. However, the collected evidence has shown that ADCs still exhibit some limitations that need to be addressed in order to optimize their efficacy/effectiveness and safety profiles [8]. Considering the aforementioned information, this review aims to provide a summarized overview of ADC pharmacotherapy, outline the current limitations restricting their optimal use, explore novel strategies addressing these challenges, and examine emerging therapeutic applications. To achieve these objectives, the methodology adopted in this article involved a bibliographic review of the available scientific literature on topics related to ADC-based targeted therapies. The information required to elaborate this review was mostly obtained from a few searchable bibliographic online databases (like PubMed^®^ and ScienceDirect^®^), books, e-books, and other kinds of documents published by international health societies and regulatory agencies. During the research phase, some of the terms inserted in the query box included “Antibody-Drug Conjugate*”, “Mechanism of Action[MeSH Terms]”, “Pharmacotherapy[MeSH Terms]”, “Pharmacovigilance[MeSH Terms]”, “Adverse Drug Reaction[MeSH Terms]”, “Limitation* OR Challenge*”, and “Future Perspective* OR Future Prospect*”.

## 2. Pharmacotherapy of ADCs

**Figure 1:**
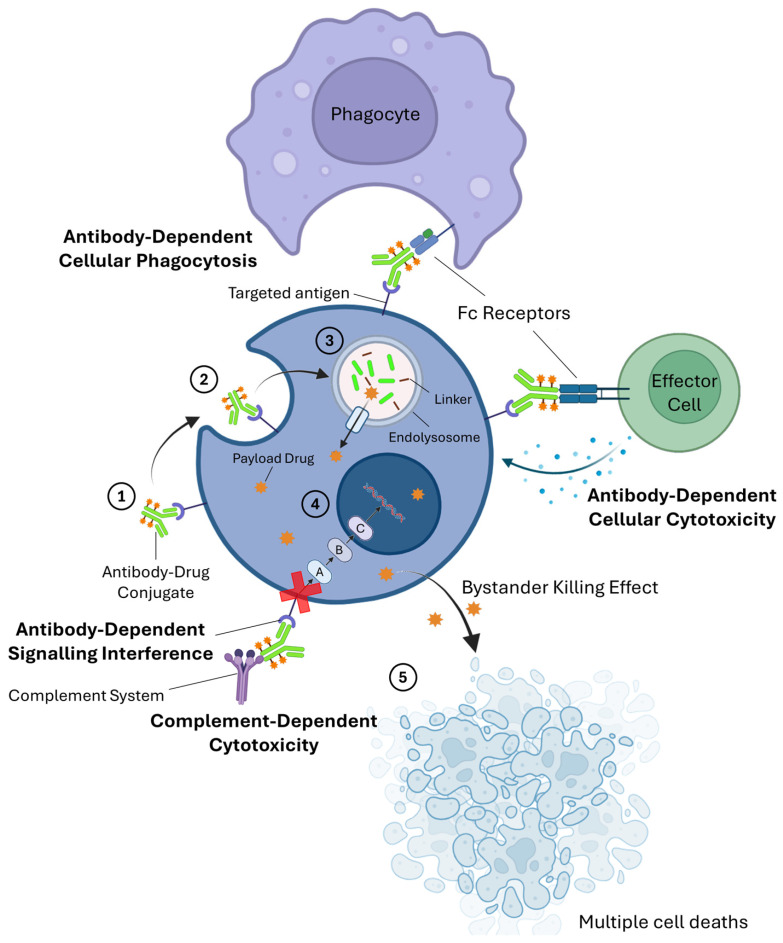
Schematic representation of the multiple mechanisms of action that antibody–drug conjugates (ADCs) can exert on tumoral cells. In addition to their main mechanism of action (the one enumerated in the figure), ADCs can also exert alternative mechanisms when they are not internalized by the targeted cell. Legend: 1—The antibody moiety of the ADC interacts with its complementary surface receptor; 2—The interaction created signals in the cell to internalize the formed complex via endocytosis; 3—The generated endosome fuses with a lysosome to promote the degradation of the internalized ADC and release of the payloads; 4—The released payloads leave the endolysosome and reach the cell cytosol where they will exert their mechanism of action on their intracellular targets or enter the nucleus, if the intracellular target is present inside this organelle; and 5—Due to the cytotoxicity of the payloads, the targeted cell undergoes cell death. When using relatively small, uncharged, and apolar payload molecules, it is sometimes observed that free payloads can easily diffuse to other adjacent cells and exert their cytotoxic mechanism of action on them, ensuring that multiple cancer cells are effectively killed, even in tumors with low internalization rates and/or heterogeneous levels of expression of the targeted surface receptor. Abbreviation: Fc, Fragment Crystallizable. Created in BioRender. Augusto, A. (2026) https://BioRender.com/hztmi6y, (accessed on 6 February 2026).

ADCs are a specialized group of biopharmaceutical products that belong to the family of targeted therapeutic agents for the treatment of cancer. As previously mentioned, they were introduced into the clinical setting to overcome the toxicity issues associated with the use of conventional chemotherapy, as well as the susceptibility for the development of drug resistance of immunotherapy with mAbs, by combining the two most favorable attributes of each of these types of cancer treatments [6]. For instance, brentuximab vedotin, an ADC used to treat stage III/IV Hodgkin’s lymphoma obtained a 2-year modified progression-free survival (PFS) of 82.1%, when compared to 77.2% with standard adriamicyn, bleomycin, vinblastine, and dacarbazine, while having a hazard ratio (HR) of 0.77 when compared to the same standard-of-care treatment [9,10]. For non-hematologic malignancies, trastuzumab emtansine, for example, extended the median overall survival (OS) to 30.9 months compared to 25.1 months with standard-of-care treatment and an HR of 0.68 in patients with human epidermal growth factor receptor 2 (HER2)-positive metastatic breast cancer [10,11]. In terms of biological activity, ADCs can exert multiple mechanisms of action. They can act similarly to mAbs by either inducing the activation of the immune system (via ADCC, ADCP, or CDC) or by blocking the signaling transduction pathways of some surface receptors involved in the proliferation of malignant cells [12]. Moreover, ADCs also possess a mechanism of action that is unique to this class of targeted therapies [13]: (i) first, the antibody moiety binds to overexpressed or specific receptors that are present on the surface of targeted cells; (ii) the interaction creates signals in the cell to internalize the newly formed ADC–antigen complex via endocytosis; (iii) the resulting endosome then fuses with a lysosome, forming an endolysosome where the internalized ADC is going to be digested, releasing the payloads from the antibody moiety through a specific release mechanism that is correlated with the type of linker used. For instance, while acid-sensitive cleavable linkers depend on the presence of lower pH values within the endolysosome to release the payloads, glutathione-sensitive disulfide linkers rely on the presence of intracellular reducing agents, such as glutathione (whose levels are typically elevated in malignant cells), to promote the release of these cytotoxic agents. Linkers can also be classified as enzymatically cleavable linkers (if they release the payloads in the presence of specific hydrolytic enzymes, particularly those that are found inside the endolysosomal lumen) or as non-cleavable linkers (if the payload release relies on the complete degradation of the antibody moiety); (iv) once released from the digested ADC, payloads leave the endolysosome and reach their specific intracellular target where they will exert their unique mechanisms of action and induce the death of targeted cells; and (v) due to their small, apolar, and uncharged molecular structure, some payloads can passively diffuse across cellular membranes, enabling them to enter the cytoplasm of adjacent tumor cells and produce a bystander killing effect, ensuring that multiple cancer cells are effectively killed, even in highly heterogeneous tumors and/or with low rates of internalization [14]. Figure 1 depicts a schematic representation of the multiple mechanisms of action that current ADCs can exert on tumoral cells. However, it does not account for several factors that can substantially impact their clinical effectiveness in real-world settings. For instance, while the figure suggests an equal (1:1) distribution between the primary and alternative mechanisms of action, in reality, only about 1–2% of administered ADCs reach, and are internalized by, the targeted tumor cells [3]. This limited uptake can be attributed to variability among tumor cells, even within the same cancer type, in terms of target antigen expression, as well as endocytic and intracellular trafficking pathways, all of which can directly affect the therapeutic response [15]. Furthermore, heterogeneity in ADC-related properties, such as the drug-to-antibody ratio (DAR, the average number of payloads per antibody moiety), can contribute to differences in clinical efficacy, as ADCs with higher DARs generally show greater therapeutic activity but also carry an increased risk of adverse effects [16].

The introduction of ADCs into the drug market was initially hindered by their structural complexity and the limited clinical benefit observed in early studies [17]. However, their clinical implementation has accelerated in recent years, partly driven by the need for improved therapeutic responses in cancers where mAb-based immunotherapies have shown limited effectiveness [18]. By the end of 2025, there was a total of twenty-one ADCs approved globally for clinical use. Of these, twenty retained their marketing authorizations at that time, while only moxetumomab pasudotox had been withdrawn from the market due to commercial reasons unrelated to its safety or efficacy. According to their therapeutic applications, ADCs can be divided into those used in hematologic malignancies or those used in non-hematologic malignancies [6]. Table 1 summarizes the most important characteristics regarding molecular composition and the main pharmacotherapeutic indications of the previously mentioned twenty-one ADCs ever approved in at least one of the major drug markets by the end of 2025. Data were extracted from both regulatory documents and the published literature. When discrepancies were identified between these two sources of information, priority was given to the most authoritative source, i.e., the regulatory documents. However, when relevant data were not available in regulatory documentation, information was supplemented from the scientific literature. Potential off-label uses were not considered. Table 2, on the other hand, summarizes the most relevant undesirable effects, drug interactions, effects on fertility, pregnancy, and lactation, alongside the most important precautions and contraindications of the ADCs previously mentioned in Table 1. The data were extracted based on the same search strategy as the one used to collect the data presented in Table 1.

## 3. Current Limitations

Although the introduction of ADCs into the clinical setting has brought several promising therapeutic advantages, particularly in relapsed, refractory, and metastatic cancers, there are still some drawbacks that need to be addressed in order to design and develop new ADCs with optimized chemical and biological properties. Some of these limitations include: (i) toxicity; (ii) susceptibility to the development of drug resistances; (iii) complex pharmacokinetic profiles; and (iv) stability issues.

Even though ADCs were conceptualized with the intent of overcoming the toxicity issues associated with the use of conventional chemotherapy (which was achieved, to a certain extent), they still retain a suboptimal safety profile, mainly attributed to the emergence of off-target toxicities related to the untimely release of payloads into the bloodstream instead of at the actual site of the tumor(s) [102]. This phenomenon is typically associated with the use of cleavable linkers that are susceptible to release of the payloads when they are exposed to certain chemical or enzymatic stimuli that are exacerbated within tumoral microenvironments and inside cancer cells but are also present in normal physiological conditions, although to a lower extent [103]. Other causes of off-target toxicity may involve the excessive bystander killing effect and the non-specific uptake of these drugs by healthy cells. For instance, the temporary withdrawal of belantamab mafodotin, an ADC bearing a non-cleavable linker, was partially associated with the excessive uptake of this drug by the corneal epithelial cells and due to the incapacity of the charged amino acid–linker–payload complex that is released from the antibody moiety to diffuse out of these cells, resulting in the emergence of frequent episodes of ocular toxicity [104]. It is worth mentioning that on-target toxicity issues may also occur with this type of targeted therapy, because until now, every antibody moiety used in currently market-approved ADCs can only target TAAs instead of TSAs [6]. In addition to the toxicity issues correlated with the cytotoxicity of the payloads used in this kind of targeted therapy, the risk of unwanted immunogenicity is another issue that is observed with the use of biologic drugs, including ADCs [105].

A major limitation associated with the use of targeted therapies is their susceptibility to drug resistances developed by cancer cells. Although ADCs were (in part) introduced into the market to overcome resistances that rapidly emerged with other treatments, such as mAb-based immunotherapies, clinical evidence indicates that resistance to ADCs can also occur [106]: (i) antigen–antibody-mediated drug resistances are a group of resistances that negatively impair the effectiveness of antibody-based therapies by reducing the expression of targeted antigens on cancer cells, increasing receptor degradation, altering receptor structure, and/or by decreasing antigen–antibody affinity [106,107,108]; (ii) upon binding the antibody moiety to its corresponding receptor, ADCs should be internalized into the targeted cell via receptor-mediated endocytosis. However, in this step, tumoral cells may develop mechanisms of decreased internalization efficiency and/or induce alterations in the endosomal transport pathways of ADCs, preventing them from exerting their expected efficacy [106,107,109]; (iii) lysosomal degradation can also contribute to drug resistance by blocking the release of payloads from ADCs and preventing their exit from the endolysosomal lumen. For example, elevated endolysosomal pH can hinder ADCs with non-cleavable and acid-labile linkers from releasing their cytotoxic agents and impede the diffusion of weakly acidic payloads into the cytosol [106]. Conversely, lowered pH may prevent hydrophobic weak-base payloads from reaching the cytosol [110]. Moreover, reduced expression of the lysosomal membrane protein solute carrier family 46 member 3 (SLC46A3) has been associated with decreased payload release from ADCs bearing non-cleavable linkers such as those derived from maytansine and SG3376 [106,111]; (iv) even if the payloads are released from the ADC and from the endolysosome, it is still possible for the targeted cell to develop resistances to the payloads themselves, similar to what is observed with the use of conventional chemotherapy. Genetic mutations, decreased expression rates of susceptible intracellular targets, increased expression rates of resistant isoforms, post-translational modifications to the targets, and upregulation of the adenosine triphosphate (ATP)-binding cassette efflux transporters are examples of payload-related drug resistance mechanisms that may arise with the use of ADCs [109,112]; (v) tumor cells can increase survival by exploiting redundancy in receptor tyrosine kinase signaling, altering cell-cycle dynamics (particularly relevant for cell-cycle dependent payloads), upregulating cytoprotective proteins, and downregulating pro-apoptotic proteins, thereby reducing the efficacy of ADCs and other cancer therapies [106,107,113]; and (vi) in addition to the resistance mechanisms that can emerge from individual cells, the tumoral microenvironment where these cells reside may also be involved in further drug resistance, as they possess immunosuppressive properties and have poor accessibility that can hinder the penetration of antibody-based therapies because of the large molecular size of these drugs and due to the elevated interstitial fluid pressure created within tumoral microenvironments, which neutralizes the pressure gradient needed for drugs to leave the bloodstream and reach the tissues [114].

In addition to the primary limitations, ADCs exhibit a complex pharmacokinetic profile that makes it difficult to predict their behavior inside the body and, consequently, adjust the dosing to each individual patient. Unlike other drugs, the pharmacokinetic profile of ADCs requires measuring not only the concentration of intact ADCs (antibody moieties and conjugated payloads) but also non-conjugated antibody moieties, unconjugated payloads, and their metabolites in the bloodstream to predict therapeutic efficacy and toxicity risk [115]. Moreover, ADCs face an increased likelihood of aggregation, even more than mAb therapies, since the hydrophobic nature of the attached payloads might facilitate the exposure of aggregation-prone regions and induce the formation of strong inter-molecular interactions with other protein-based drugs [116]. The formed aggregates not only prevent the sequestered molecules from binding to their intended targets and exerting their expected mechanism of action; however, they can also trigger unwanted immune reactions and increase the risk of off-target payload release into the bloodstream, reducing efficacy and raising safety concerns at the same time with the use of ADCs [116].

## 4. Novel Approaches to the Concept of ADC and New Therapeutic Applications

The introduction of ADCs revolutionized targeted therapies for cancer by leveraging the selectivity of mAbs to deliver potent cytotoxic agents directly to cancer cells, offering therapeutic advantages over other treatments. However, clinical use has revealed limitations that must be addressed to enhance the efficacy and safety of future ADCs. Key proposed improvements for next-generation ADC design and development include the following aspects [117]: (i) better tumor penetration capacity; (ii) improved microenvironment targeting activity, instead of just targeting individual tumoral cells; (iii) flexibility to comprise novel payloads with new mechanisms of action and innovative approaches that promote enhanced activity against tumoral cells, while causing minimal damages to healthy cells; (iv) adjustable linker chemistry and its conjugation techniques, which should be improved to prevent the premature discharge of payloads into the bloodstream, whilst facilitating their release inside cancer cells or within tumoral microenvironments; (v) reduced risk of unwanted immunogenicity and on-target toxicity caused by the antibody moiety; and (vi) enhanced internalization trafficking and capacity to target multiple and, preferably, highly conserved cellular structures within tumoral cells.

**Figure 2:**
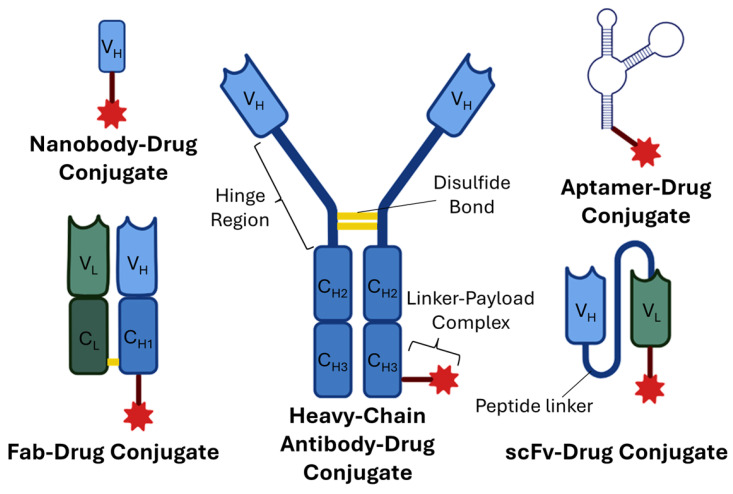
Schematic representation of the novel approaches designed to overcome the limitations associated with the poor penetration capacity of market approved antibody–drug conjugates to the tumoral microenvironment of solid tumors. Abbreviations: C_H1_, Constant domain 1 of the heavy chain; C_H2_, Constant domain 2 of the heavy chain; C_H3_, Constant domain 3 of the heavy chain; C_L_, Constant domain of the light chain; Fab, Fragment antigen-binding; scFv, Single-chain variable fragment; V_H_, Variable domain of the heavy chain; and V_L_, Variable domain of the light chain. Created in BioRender. Augusto, A. (2026) https://BioRender.com/bl37z7p, (accessed on 14 February 2026).

As was previously mentioned, one of the main limitations with the use of large biomolecules in the treatment of solid tumors is their accessibility, especially to deeper cells within the tumoral microenvironments. Instead of using a fully assembled antibody moiety, it is possible to enhance the penetration capacity of ADCs by using smaller molecules or antibody derivatives that can still interact with similar selectivity to their complementary targets, when compared to their fully assembled counterparts [13]. Furthermore, using smaller molecules or antibody fragments can significantly reduce the production cost and complexity of these types of drugs, as they could be produced in microbial expression systems instead of using mammalian expression systems, allowing for the fragments to be produced more quickly and at higher yield rates [118]. In addition, smaller molecules (e.g., aptamers, peptides, and other small-molecule linkers) or antibody fragments can substantially decrease the potential risk of unwanted immunogenicity, as they possess fewer domains recognizable by the immune system [118,119]. However, smaller formats can also exhibit altered pharmacokinetics, including reduced circulation half-life; the absence of a fragment crystallizable (Fc) region in these fragments prevents the activation of immune effector functions (e.g., ADCC, ADCP, and CDC) [120,121]. Examples of some novel alternatives to ADCs using antibody fragments and smaller molecules include the fragment antigen-binding (Fab)-drug conjugate (a type of ADC comprising just one of two Fab regions of an antibody, instead of using the entire molecular structure of the antibody), the single-chain variable fragment (scFv)–drug conjugate (a conjugate-based drug incorporating a fragment containing the variable region of one heavy and one light chain connected by a peptide linker with 15–20 amino acids), the nanobody–drug conjugate (a small conjugated drug that uses the variable domain of a single antibody heavy chain instead of the entire antibody), and the aptamer–drug conjugate (which, instead of using an antibody derivative, uses a single-stranded oligonucleotide that folds on its own to acquire a unique three-dimensional structure, enabling it to bind to specific targets with high affinity and specificity) [122,123]. Another approach is the use of heavy-chain ADCs, where the antibody moiety is modified to lack the two light chains and the first constant domain of each heavy chain. While this novel alternative shows limited improvement in penetration capacity, compared to other drug conjugation strategies, heavy-chain ADCs are unique in their ability to activate the immune system, given the presence of the Fc region [124]. Figure 2 shows a schematic representation of the novel approaches that have been considered to overcome the poor penetration capacity of ADCs in solid tumors.

**Figure 3:**
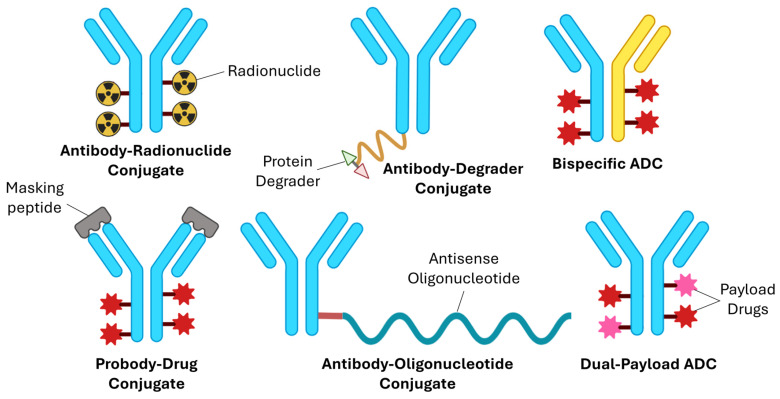
Schematic representation of some of the strategies that are being considered for the development of new antibody–drug conjugates with improved efficacy, better capacity to overcome drug resistance, and higher safety profiles. Abbreviations: ADC, Antibody–drug conjugate. Created in BioRender. Augusto, A. (2026) https://BioRender.com/31ofzwe, (accessed on 14 February 2026).

Tumoral microenvironments are known to be enriched with various types of proteases, such as matrix metalloproteases and serine proteases, that can be used to enhance the selectivity of antibody moieties that target TAAs [125]. One such strategy involves protecting the antigen-binding sites of the antibody moiety with small masking peptides that are covalently bound to the antibody on each antigen-binding site through a cleavable linker sensitive to these proteases [126]. Upon reaching the protease-enriched medium of tumoral microenvironments, the protease-sensitive linkers are cleaved, and the masking peptides are released from the antibody, restoring the availability of the antigen-binding sites. This allows probody–drug conjugates (Figure 3) to remain inactive in circulation, preventing them from interacting with the membrane receptors of healthy cells, ensuring that the probody is only going to be bioactivated near tumoral cells [126].

Future improvements to antibody moieties in ADCs should focus on enhancing the penetration of large biomolecules, while optimizing the overall therapeutic efficacy and safety of this component. For instance, by using bispecific antibodies (Figure 3) it is possible to overcome some safety concerns related to on-target toxicity, as they can target two distinct TAAs that, ideally, are not present simultaneously on healthy cells but coexist on tumor cells, enabling selective internalization or activation of the immune system only when both antigen-binding sites interact with their respective targets [127]. Antibody moieties that interact with highly conserved structures that are less likely to undergo molecular alteration have a lower probability to fall prey to the development of drug resistance [128]. Engineering the Fc region can also be considered to enhance Fc-mediated effectors functions and to optimize the structure of the antibody moiety, so the latter can act as an independent drug. Therefore, the original ADC becomes less dependent on the cytotoxicity of the small fraction of payloads that are internalized to exert an effective therapeutic response [129]. Beyond what was previously stated, the use of human-derived antibodies should be taken into account to reduce the risk of unwanted immunogenicity associated with the use of non-human antibodies [117].

Most adverse drug reactions associated with ADCs arise from the on- and off-target cytotoxicity of payloads to healthy cells. On-target toxicity is primarily due to antibody moieties binding to TAAs on healthy cell membranes, while off-target toxicity is often linked to linker instability, leading to premature payload release into the bloodstream. Additionally, payload chemistry plays a significant role [103]. Excessive hydrophobicity can increase aggregation, immunogenicity, and unwanted diffusion to healthy cells, while high hydrophilicity may prevent effective bystander killing of receptor-negative cancer cells [103]. In addition, novel payloads need to overcome drug resistance [106]. Some of the most innovative developments proposed for new payloads include the use of multiple payloads with different intracellular mechanisms of action and the exploitation of new types of payloads, such as, for example, immune stimulator compounds used as agonists of stimulator of interferon genes (STING) and toll-like receptors (TLRs), two crucial receptors involved in the activation of the innate immune system [130]. The use of antisense oligonucleotides (short sequences of single-stranded deoxyribonucleic acid (DNA) or ribonucleic acid (RNA) designed to bind to specific messenger RNA (mRNA) sequences in order to alter the mRNA involved in the pathogenesis of the disease and modulate protein expression through several distinct biological mechanisms) have also been considered, as well as the use of radionuclides and protein degraders such as proteolysis targeting chimeras (PROTACs, a type of heterobifunctional molecules containing a protein-of-interest binding domain connected to an E3 ubiquitin ligand by a linker. PROTACs target specific proteins to be degraded by taking advantage of the ubiquitin–protease system to bring together the target protein and an E3 ligase) [131,132,133]. Figure 3 provides a schematic representation of some of these novel approaches to the development of future payloads.

**Figure 4:**
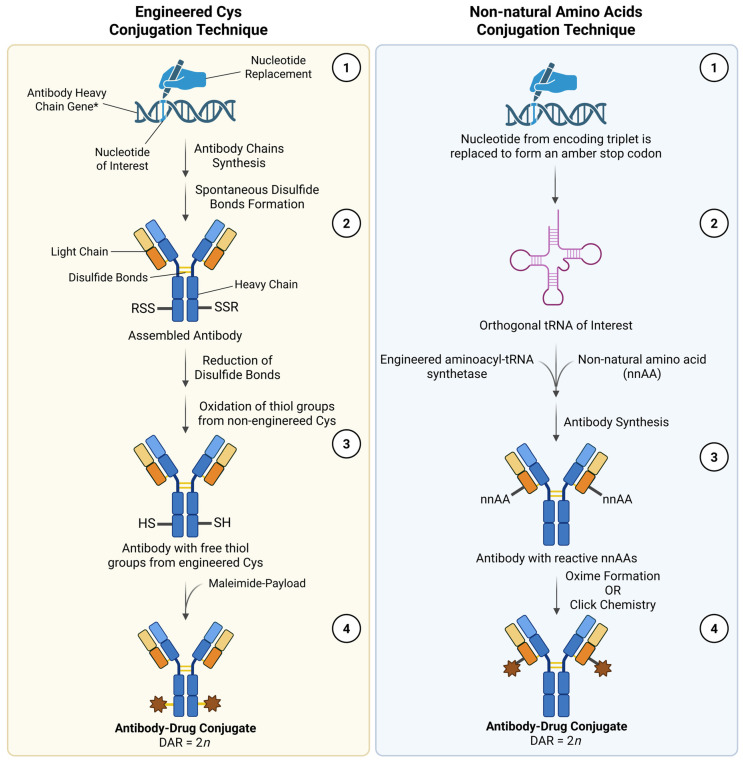
Emerging site-specific ADC conjugation techniques with engineered cysteines (on the left) and non-natural amino acids (on the right). Engineered cysteines conjugation technique: (1) A number (*n*) of single nucleotide replacements are made in the heavy- and/or light-chain genes that encode the antibody moiety to promote synthesis of a modified antibody with 2*n* strategically introduced cysteine residues at predetermined positions. Upon being synthesized, the antibody chains spontaneously assemble the antibody structure through the formation of disulfide bonds between the free thiol groups of natural cysteines present within the molecular structure of the antibody chains. During this process, engineered cysteines also form disulfide bonds with other free thiol groups (–SSR); (2) In order to make it possible for 2*n* payloads conjugate with the antibody moiety, the engineered cysteines must to be reduced and the natural ones need to be maintained as oxidized; (3) Then, the desired payload carrying a reactive maleimide group is added into the reactional medium to promote the conjugation of the ADC components; (4) The newly formed ADCs are expected to possess a DAR equal to two times the number of nucleotide replacements that led to the substitution of a natural amino acid with an engineered cysteine. Non-natural amino acid conjugation technique: (1) A nucleotide from an encoding triplet is replaced with another one that leads the formation of an amber stop codon (UAG, i.e., uracil–adenine–guanine); (2) An orthogonal transfer RNA (tRNA) specific for the amber stop codon is submitted to the reaction catalyzed by an engineered aminoacyl-tRNA synthetase, which selectively charges the tRNA with a desired non-natural amino acid (nnAA); (3) Upon the formation of the modified antibody, the engineered nnAAs become readily available to react with the specialized and highly reactive groups of desired payloads; (4) The newly formed ADCs, similar to what is observed with the conjugation technique with engineered cysteines, possess a DAR equal to 2*n*. Legend: Cys, Cysteine; DAR, Drug-to-antibody ratio; *n*, Number of nucleotide replacements; nnAA, Non-natural amino acid; RSS or SSR, Disulfide bond between the engineered cysteine and other molecules with free thiol groups; SH, Free thiol group; tRNA, Transfer ribonucleic acid. Please note that, for schematic purposes, it was considered that the conjugation site of engineered cysteines and non-natural amino acid conjugation processes was within the heavy and light chain, respectively. However, it could be the other way around. It will depend on the gene where the nucleotide replacement was made. Adapted from [137,138,139]. Created in BioRender. Augusto, A. (2026) https://BioRender.com/zumgxye, (accessed on 5 April 2026).

Finally, the linker chemistry is another critical factor involved in both the safety and efficacy of ADCs. As mentioned earlier, premature off-target release, caused by poor linker stability, is a major factor in ADC toxicity. However, the aggregation phenomena that is associated with decreased serum stability is closely correlated with the conjugation technique used to bind the payloads to the antibody moiety [134]. For instance, while low DARs are linked to reduced therapeutic outcomes, ADCs with high DARs tend to exhibit stability issues, potentially resulting in the premature release of conjugated payloads, increasing the risk of off-target toxicity [6]. To address the limitations of current linkers, it is important to improve their stability in solution and ensure a rapid payload release only when they are exposed to unique chemical or enzymatic triggers solely found inside cancer cells and within their microenvironments. Additionally, innovative controlled homogenous conjugation techniques can help to produce more uniform molecules with better DAR values and a more predictable pharmacokinetic behavior, while maintaining the antibody’s integrity [135]. Examples of such techniques include the engineered amino acid approaches (which use engineered cysteines and non-natural amino acids) (Figure 4), enzyme-mediated approaches (with transglutaminase, formylglycine-generating enzyme, sortase A, glycotransferases, and endoglycosidase), and linker-based approaches (with dibromopyridazinediones, dibromomaleimides and other enhanced maleimides, with hydrophilic linkers, and/or with bis-alkylating linkers) [136]. Figure 4 presents a simplified diagram of the reactional steps that need to be conducted in order to produce new ADCs with homogeneous DARs through engineered amino acid approaches. While enzymatic and linker-based conjugation strategies are increasingly prominent in industrial pipelines, site-specific conjugation techniques with engineered cysteine and non-natural amino acid are presented here as representative models due to their conceptual clarity.

**Figure 5:**
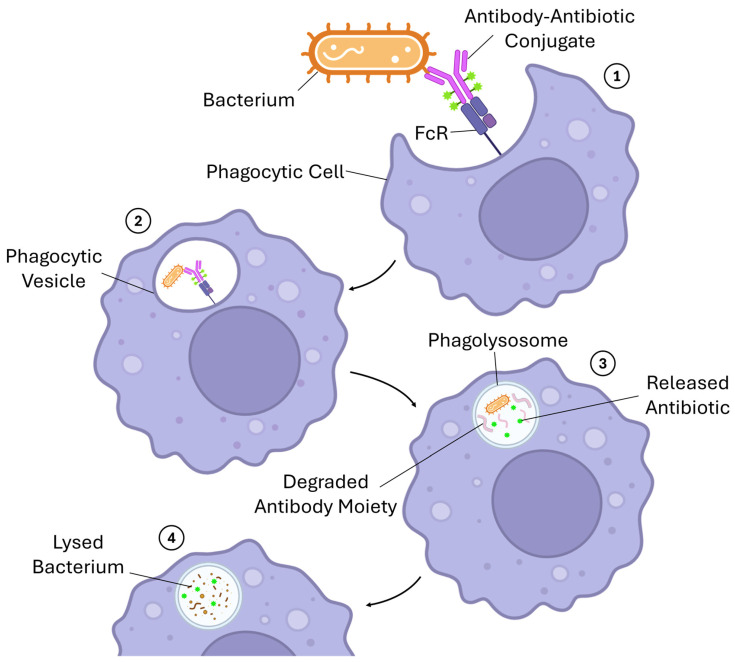
Proposed mechanism of action of antibody–antibiotic conjugates, a new therapeutic approach that facilitates the activation of the immune system against severe bacterial infections that failed previous treatments with unconjugated antibiotics. Legend: 1—The antibody–antibiotic conjugate interacts with a targeted antigen present on the surface of the bacterium through its specific antibody moiety. Upon interaction, phagocytic cells recognize the exposed Fc region of the antibody moiety through their complementary Fc receptor present on the surface of effector cells. Following the recognition, the phagocytic engulfs the opsonized bacterium (opsonization is not shown in the figure); 2—The newly formed phagocytic vesicle fuses with a lysosome to promote the degradation of the pathogen; 3—During the degradation step, proteolytic enzymes also digest the antibody moiety, allowing for the conjugated antibiotics to be released; and 4—The released antibiotics aid the immune system eliminate the pathogens by exerting their specific mechanism of action on the targeted bacterium. Abbreviations: FcR, Fragment crystallizable receptor. Adapted from [142]. Created in BioRender. Augusto, A. (2026) https://BioRender.com/q32cuub, (accessed on 19 February 2026).

Given the success of ADCs, researchers have already proposed expanding their therapeutic applications [6]. While most ADCs are in clinical trials for other stages and cancer types, a few ADC candidates are also being explored for non-oncological conditions such as inflammatory diseases and infectious diseases (especially, against *Staphylococcus aureus* infectious) [140]. Although not yet in clinical trials, the use of ADCs in neurodegenerative diseases has also been proposed and is under development [141]. Notwithstanding the fact that all the non-oncological clinical trials form only around 1% of the currently running clinical trials involving ADCs, the potential use of this type of targeted therapy in the future for some diseases beyond cancer is bound to mark one of the biggest advancements in the history of targeted therapies [141]. Figure 5 illustrates the unique mechanism of action proposed for antibody–antibiotic conjugates, the ADC-derivative approach that uses antibiotics as payloads instead of cytotoxic agents.

## 5. ADCs in Clinical Trials

Table 3 shows examples of ADCs in phase I–III clinical trials identified in the Clinicaltrials.gov database, indicating targets, payload, condition/disease, and other important observations. Several targets for ADCs are highlighted such as human epidermal growth factor receptor 2 (HER2), trophoblast cell surface antigen 2 (TROP2), human epidermal growth factor receptor 3 (HER3), and claudin 18.2 (CLDN18.2).

## 6. Conclusions

The history of ADCs is a remarkable example of how precision pharmacotherapy has evolved since the day Paul Ehrlich proposed the concept of “magic bullets”. Although ADCs faced a challenging beginning, they are now believed to have changed the paradigm regarding targeted therapies, a type of treatment that takes advantage of the presence of cell surface receptors to promote the selective delivery of potent cytotoxic agents inside the cytoplasm of cancer cells, while at the same time minimizing unwanted damage to healthy cells.

The introduction of ADCs into the therapeutic drug arsenal available against oncological diseases (hematologic and non-hematologic) has led to significant improvements in the treatment of some types of cancer, specifically in advanced or metastatic stages. However, their clinical use still faces some limitations and challenges that need to be overcome to optimize their therapeutic potential. Several improvements to the target selection and the characteristics of ADC components have been proposed; however, further investigation is still needed. The overall great success achieved with the introduction of ADCs into the drug market has sparked significant interest regarding the expansion of their therapeutic applications to new diseases beyond cancer, such as inflammatory, infectious, and neurodegenerative diseases.

## Figures and Tables

**Table 1:** Molecular composition and main pharmacotherapeutic indications of most clinically relevant antibody–drug conjugates worldwide. Abbreviations: AcBut, 4-(4-Acetylphenoxy)butanoic acid; Ala, Alanine; BCMA, B-Cell Maturation Antigen; CD, Cluster of Differentiation; Cit, Citrulline; c-Met, Cellular Mesenchymal-Epithelial Transition factor; DAR, Drug-to-Antibody Ratio; DM1, Mertansine; DM4, Ravtansine; EGFR, Epidermal Growth Factor Receptor; Frα, Folate Receptor alpha; Gly, Glycine; HER2, Human Epidermal growth factor Receptor 2; HR, Hormone Receptor; IgG1, Immunoglobulin G1; IgG4, Immunoglobulin G4; INN, International Non-proprietary Name; Mal, Maleimide; Mc, Maleimidocaproyl; MCC, 4-(*N*-maleimidomethyl)cyclohexane-1-carboxylate; MMAE, Monomethyl Auristatin E; MMAF, Monomethyl Auristatin F; NSCLC, Non-Small Cell Lung Cancer; PABC, para-Aminobenzyl Carbamate; PD-1, Programmed Death protein 1; PD-L1, Programmed Death-Ligand 1; PEG, Polyethylene Glycol; Ph^+^, Chromosome Philadelphia-positive; Phe, Phenylalanine; TF, Tissue Factor; TNBC, Triple-Negative Breast Cancer; Trop2, Trophoblast Cell-Surface Antigen 2; and Val, Valine.

INN	Antibody Moiety	Linker	Payload	DAR	Therapeutic Indication(s)	Reference
**Antibody–Drug Conjugates used in Hematologic Malignancies**						
**Belantamab mafodotin**	Anti-BCMA IgG1	Maleimido-Caproyl (non-cleavable)	MMAF (Antimitotic Agent)	4	Adult patients with relapsed or refractory multiple myeloma (combination therapy)	[19,20]
**Brentuximab vedotin**	Anti-CD30 IgG1	Mc-Val-Cit-PABC (protease-sensitive)	MMAE (Antimitotic Agent)	4	First-line treatment of adult patients with stage III/IV CD30+ Hodgkin lymphoma (combination therapy). It can also be used in adult patients with systemic anaplastic large cell lymphoma (in combination) or with CD30+ cutaneous T-cell lymphoma (monotherapy)	[9,21]
**Gemtuzumab ozogamicin**	Anti-CD33 IgG4	AcBut (acid, and glutathione-sensitive)	*N*-Acetyl-Calicheamicin γ_1_ ^I^ (DNA Damaging Agent)	2–3	Patients aged 15 years or above, with previously untreated *de novo* CD33+ core binding factor acute myeloid leukemia (combination therapy)	[22,23]
**Inotuzumab ozogamicin**	Anti-CD22 IgG4	AcBut (acid and glutathione-sensitive)	*N*-Acetyl-Calicheamicin γ_1_ ^I^ (DNA Damaging Agent)	6	Adult patients with relapsed or refractory CD22+ (first line) and Ph^+^ (second line) B cell precursor acute lymphoblastic leukemia (monotherapy)	[24,25]
**Loncastuximab tesirine**	Anti-CD19 IgG1	Mal-PEG8-Val-Ala-PABC (protease-sensitive)	SG3199 (DNA Damaging Agent)	2.3	Adult patients with relapsed or refractory diffuse large B-cell lymphoma and high-grade B-cell lymphoma (monotherapy)	[26,27]
**Moxetumomab pasudotox** (Not authorised)	Anti-CD22 IgG1	Mc-Val-Cit-PABC (protease-sensitive)	Truncated form of *Pseudomonas* exotoxin A (Protein Synthesis Inhibitor)	1	Adult patients with relapsed or refractory hairy cell leukemia after receiving at least two prior systemic therapies (monotherapy)	[28,29]
**Polatuzumab vedotin**	Anti-CD79b IgG1	Mc-Val-Cit-PABC (protease-sensitive)	MMAE (Antimitotic Agent)	3–4	Adult patients with previously untreated diffuse B-cell lymphoma or with relapsed/refractory diffuse B-cell lymphoma who cannot undergo transplantation (combination therapy)	[30,31]
**Antibody–Drug Conjugates used in Non-Hematologic Malignancies**						
**Becotatug vedotin**	Anti-EGFR IgG1	Mc-Val-Cit-PABC (protease-sensitive)	MMAE (Antimitotic Agent)	3.8	Adult patients with recurrent or metastatic nasopharyngeal carcinoma	[32,33]
**Cetuximab sarotalocan**	Anti-EGFR IgG1	Linear alkyl/alkoxy linker (non-cleavable)	IRDye 700DX (Near-Infrared Photosensitizer)	Between 1.3–3.8	Adult patients with unresectable locally recurrent head and neck squamous cell carcinoma	[34,35]
**Datopotamab deruxtecan**	Anti-Trop2 IgG1	Mc-Gly-Gly-Phe-Gly (protease-sensitive)	DXd (Topoisomerase I Inhibitor)	4	Adult patients with unresectable or metastatic HR+, HER2− breast cancer who have received other treatments (monotherapy)	[36,37]
**Disitamab vedotin**	Anti-HER2 IgG1	Mc-Val-Cit-PABC (protease-sensitive)	MMAE (Antimitotic Agent)	4	Adult patients with HER2-overexpressed locally advanced or metastatic gastric cancer or urothelial carcinoma. It can also be used for HER2+ and for HER2-low-expressing metastatic breast cancer with liver metastases (monotherapy)	[38,39]
**Enfortumab vedotin**	Anti-Nectin-4 IgG1	Mc-Val-Cit-PABC (protease-sensitive)	MMAE (Antimitotic Agent)	3.8	First-line treatment for adult patients with advanced or metastatic urothelial carcinoma who are eligible for platinum-containing chemotherapy (combination therapy). It can also be used in adult patients with locally advanced or metastatic urothelial cancer who have received prior platinum-containing chemotherapy and a PD-1 or a PD-L1 inhibitor (monotherapy)	[40,41]
**Mirvetuximab soravtansine**	Anti-FRα IgG1	Sulfo-TBA (glutathione-sensitive)	DM4 (Antimitotic Agent)	3.4	Alternative treatment of adult patients with FRα+, platinum-resistant high-grade serous epithelial ovarian, fallopian tube, or primary peritoneal cancer (monotherapy)	[42,43]
**Sacituzumab govitecan**	Anti-Trop2 IgG1	CL2A (acid-sensitive)	SN-38 (Topoisomerase I Inhibitor)	7.6	Adult patients with unresectable or metastatic TNBC or HR+, and HER2- breast cancer (monotherapy)	[44,45]
**Sacituzumab tirumotecan**	Anti-Trop2 IgG1	Pyrimidine-CL2A-carbonate (acid-sensitive)	KL610023 (Topoisomerase I Inhibitor)	7.4	Adult patients with unresectable locally advanced or metastatic TNBC and metastatic HR+/HER2- breast cancer (monotherapy). It can also be used in EGFR-mutant locally advanced or metastatic non-squamous NSCLC following tyrosine kinase inhibitors and platinum-based chemotherapy (monotherapy)	[46,47]
**Telisotuzumab vedotin**	c-Met-directed IgG1	Mc-Val-Cit-PABC (protease-sensitive)	MMAE (Antimitotic Agent)	3.1	Adult patients with locally advanced or metastatic, NSCLC with high c-Met protein overexpression (monotherapy)	[48,49]
**Tisotumab vedotin**	Anti-TF IgG1	Mc-Val-Cit-PABC (protease-sensitive)	MMAE (Antimitotic Agent)	3–4	Adult patients with recurrent or metastatic cervical cancer with disease progression on or after systemic therapy (monotherapy)	[50,51]
**Trastuzumab botidotin**	Anti-HER2 IgG1	K-lock-Val-Cit-PABC (protease-sensitive)	Duostatin 5 (Antimitotic Agent)	2	Adult patients with unresectable or metastatic HER2+ breast cancer who have received one or more prior anti-HER2 therapies (monotherapy)	[52,53]
**Trastuzumab deruxtecan**	Anti-HER2 IgG1	Mc-Gly-Gly-Phe-Gly (protease-sensitive)	DXd (Topoisomerase I Inhibitor)	8	Second-line treatment for adult patients with HER2+ metastatic breast cancer with stable or undetectable brain metastases after disease progression or with active brain metastases who cannot undergo local intervention (monotherapy). It can also be used in adult patients with advanced HER2+ non-small cell lung cancer or with advanced HER2+ gastric cancer or gastroesophageal junction adenocarcinoma (monotherapy)	[54,55]
**Trastuzumab emtansine**	Anti-HER2 IgG1	MCC (non-cleavable)	DM1 (Antimitotic Agent)	3.5	Adjuvant treatment of adult patients with HER2+ early breast cancer with residual invasive disease (monotherapy). It can also be used as a second- or third-line alternative to HER2+ metastatic breast cancer in adult patients with non-existent or stabilized brain metastases (monotherapy)	[56,57]
**Trastuzumab rezetecan**	Anti-HER2 IgG1	Mc-Gly-Gly-Phe-Gly (protease-sensitive)	SHR9265 (Topoisomerase I Inhibitor)	5.7	Adult patients with HER2-mutant NSCLC (monotherapy)	[58,59]

**Table 2:** Undesirable effects, drug interactions, influence on fertility, pregnancy and lactation, as well as the key precautions and contraindications of the most clinically relevant antibody–drug conjugates worldwide. Abbreviations: CYP3A4, Cytochrome P450 3A4; CYP3A5, Cytochrome P450 3A5; G-CSF, Granulocyte colony-stimulating factor; HUS, Hemolytic uremic syndrome; ILD, Interstitial lung disease; INN, International non-proprietary name; N/A, Not applicable; SJS, Stevens–Johnson syndrome; TLS, Tumor lysis syndrome; UGT1A1, Uridine diphosphate-glucuronosyl transferase; and VOD/SOS, Veno-occlusive disease/Sinusoidal obstruction syndrome.

INN	Undesirable Effects	Drug Interactions	Fertility, Pregnancy, and Lactation	Special Precautions and Contraindications	Reference
**Antibody–Drug Conjugates used in Hematologic Malignancies**					
**Belantamab mafodotin**	Ocular toxicity, neutropenia, anemia, diarrhea, neuropathies, pneumonia, pyrexia and reactivation of Hepatitis B	N/A	Limited data. Usually, not recommended for use during pregnancy neither during breastfeeding. May cause reproductive and embryo–fetal toxicity	Regular ophthalmologic monitoring recommended. Patients should be advised to avoid driving or operating heavy machinery, when visual acuity is affected	[60,61]
**Brentuximab vedotin**	Pulmonary toxicity, progressive multifocal leukoencephalopathy, pancreatitis, serious and opportunistic infections, TLS, peripheral neuropathy, hematological toxicities, severe cutaneous adverse reactions, gastrointestinal complications, hepatotoxicity and hyperglycemia	Inhibitors and inducers of CYP3A4	Limited data. Usually, not recommended for use during pregnancy neither during breastfeeding. May cause reproductive toxicity	Combined use with bleomycin is contraindicated, due to increased risk of pulmonary toxicity	[62,63]
**Gemtuzumab ozogamicin**	Hepatotoxicity (including VOD/SOS), hemorrhage, risk of infection, TLS, myelosuppression, pyrexia and tachycardia	Minor interactions. Not clinically significant	Limited data. Usually, not recommended for use during pregnancy neither during breastfeeding. May cause reproductive toxicity	Due to elevated risk of infections and hemorrhagic reactions, patients should undergo complete blood count prior to each administration	[64,65]
**Inotuzumab ozogamicin**	Hepatotoxicity (especially, VOD/SOS), myelosuppression, QT interval prolongation, TLS and increased amylase and lipase	Drugs that increase QT interval	Limited data. Usually, not recommended for use during pregnancy neither during breastfeeding	Contraindicated use on patients with prior VOD/SOS or serious ongoing hepatic diseases. Concomitant use with other drugs that prolong QT interval should be carefully assessed, due to the increased risk for torsade de pointes	[66,67]
**Loncastuximab tesirine**	Effusion and oedema, myelosuppression, risk of infections, photosensitivity and cutaneous reactions	Minor interactions. Not clinically significant	Testicular and embryo–fetal toxicity. General use is not recommended	Serious effusion, edema, serious or severe myelosuppression, fatal and serious infections, and serious cutaneous reactions have been reported. Complete blood cell counts should be monitored prior to each dose	[68,69]
**Moxetumomab pasudotox** (Not authorised)	HUS, CLS, hypoalbuminemia, nausea, edema, infusion related reactions, increased transaminases and/or blood creatinine	N/A	Maternal and embryo–fetal toxicity when administered in pregnant woman	Contraindicated use in patients with pre-existing severe renal impairment (creatinine clearance ≤ 29 mL/min)	[70,71]
**Polatuzumab vedotin**	Myelosuppression, risk of infections, TLS, hepatotoxicity, peripheral neuropathy, and progressive multifocal leukoencephalopathy	Inhibitors and inducers of CYP3A4	Testicular and embryo–fetal toxicity. General use is not recommended	Neutropenia, severe and severe febrile neutropenia, severe infections, and peripheral neuropathy have been reported. Blood cell counts should be determined before each dose	[72,73]
**Antibody–Drug Conjugates used in Non-Hematologic Malignancies**					
**Becotatug vedotin**	Myelosuppression, elevated transaminases, skin rash, hair loss, itching, decreased sensation, decreased appetite, myalgia, weight loss, weakness, constipation, intestinal obstruction, peripheral neuropathy, and pneumonia	Inhibitors and inducers of CYP3A4	Limited data. Usually, not recommended for use during pregnancy neither during breastfeeding. May cause reproductive toxicity	Patients should be monitored for the emergence or worsening of myelosuppression signs and symptoms, especially for febrile neutropenia	[74,75]
**Cetuximab sarotalocan**	Arterial and tumor hemorrhage, swollen tongue, laryngeal oedema, infusion reaction, photosensitivity, severe skin disorders, fatigue, dysphagia, constipation, hyponatremia, and tumor pain	Limited data. May interact with photosensitizing agents and drugs that cause hypomagnesaemia	Limited data. May induce miscarriage or embryonic death. Usually, not recommended during pregnancy	Contraindicated in patients with tumor invasion in the carotid artery. Only patients considered eligible may receive this therapy	[76,77]
**Datopotamab deruxtecan**	Interstitial lung disease or pneumonitis, keratitis and stomatitis	Minor interactions. Not clinically significant	Causes embryo–fetal toxicity. General use is not recommended	Patients may need to consider undergoing preventive treatment prior to the infusion to prevent the occurrence of infusion related reactions	[78,79]
**Disitamab vedotin**	Gastrointestinal issues, fever, fatigue, peripheral sensory neuropathy (including hypoesthesia), hematologic toxicity (including neutropenia and leukopenia), increased aminotransferases and conjugated blood bilirubin	Inhibitors and inducers of CYP3A4	Embryo–fetal toxicity and impair fertility. General use is not recommended	Patients should be monitored for the emergence of hematological abnormalities, peripheral neuropathy, or liver disfunction	[80,81]
**Enfortumab vedotin**	Skin reactions, pneumonitis or interstitial lung disease, hyperglycemia, severe infections, peripheral neuropathy and ocular disorders	Inhibitors and inducers of CYP3A4	Embryo–fetal toxicity. General use is not recommended. May cause testicular toxicity	Patients should be monitored for eye conditions	[82,83]
**Mirvetuximab soravtansine**	Ocular disorders, pneumonitis, peripheral neuropathy, nausea and vomiting	Inhibitors and inducers of CYP3A4	Embryo–fetal toxicity. General use is not recommended	Patients may need to consider undergoing preventive treatment prior to the infusion to prevent the occurrence of nausea and vomiting and/or infusion related reactions	[84,85]
**Sacituzumab govitecan**	Neutropenia, severe diarrhea, nausea and vomiting and life-threatening hypersensitivity reactions	Inhibitors and inducers of UGT1A1	Embryo–fetal toxicity. General use is not recommended	Loperamide should be used to reduce the severity of diarrhea. Patients may need to consider undergoing preventive treatment prior to the infusion to prevent the occurrence of nausea and vomiting and/or hypersensitivity side effects. Genetic variants of UGT1A1 have an increased risk of higher exposure to SN-38	[86,87]
**Sacituzumab tirumotecan**	Neutropenia, anemia, leukopenia, diarrhea, nausea, and fatigue	Inhibitors and inducers of UGT1A1	Teratogenicity and/or embryo–fetal lethality. It might cause serious adverse reactions in a breastfed child. Generally, use is not recommended	Treatment should be withheld for absolute neutrophil count below 1500/mm^3^ or neutropenic fever. Administration of G-CSF for secondary prophylaxis may be considered. Patients with diarrhea should be monitored and, if needed, treated for fluid and electrolyte imbalances	[88,89]
**Telisotuzumab vedotin**	Peripheral neuropathy, ILD/pneumonitis, ocular surface disorders, and infusion-related reactions	Inhibitors and inducers of CYP3A4	Embryo–fetal toxicity and impair fertility. General use is not recommended	Patients should be monitored for the appearance or worsening of signs and symptoms associated with the undesirable effects	[90,91]
**Tisotumab vedotin**	Ocular toxicity, peripheral neuropathy and severe cutaneous adverse reactions (including SJS)	Inhibitors and inducers of CYP3A4	Embryo–fetal toxicity. General use is not recommended	Patients should be monitored for the appearance or worsening of ocular signs and symptoms	[92,93]
**Trastuzumab botidotin**	Corneal disorders, dry eye, and blurred vision	Limited data but may be sensitive to CYP3A4 inhibitors and inducers	Limited data. General use is not recommended	Patients should be monitored for the appearance or worsening of ocular signs and symptoms	[94,95]
**Trastuzumab deruxtecan**	Pneumonitis or ILD, neutropenia and left ventricular dysfunction	Minor interactions. Not clinically significant	Embryo–fetal toxicity. General use is not recommended	Patients should be monitored with complete blood counts and for signs and symptoms of ILD/pneumonitis	[96,97]
**Trastuzumab emtansine**	Thrombocytopenia, hemorrhage, hepatotoxicity, neurotoxicity, left ventricular dysfunction and pulmonary toxicity	Inhibitors and inducers of CYP3A4 and CYP3A5	Limited data. Usually not recommended for use during pregnancy neither during breastfeeding	Monitoring of platelet count and liver function is recommended. Patients should be carefully observed for the appearance of hypersensitivity/allergic reactions	[98,99]
**Trastuzumab rezetecan**	Leukopenia (especially, neutropenia), anemia, thrombocytopenia, and ILD	Minor interactions. Not clinically significant	Carries significant risks to fetal development. A negative pregnancy test is required within 7 days before starting the treatment. Use is generally not recommended	Requires close monitoring for serious treatment-related adverse events, especially for ILD/pneumonitis and hematologic toxicity	[100,101]

**Table 3:** Names and other important characteristics of antibody–drug conjugates currently in clinical trials. Abbreviations: CDH3, Cadherin-3; CDH17, Cadherin-17; CEACAM5, Carcinoembryonic antigen-related cell adhesion molecule 5; CLDN6, Claudin 6; CLDN18.2, Claudin 18.2; c-Met, Mesenchymal–epithelial transition factor; DLK1, Delta-like 1 homolog; DLL3, Delta-like ligand 3; DNA, Deoxyribonucleic acid; EGFR, Epidermal growth factor receptor; FAP-alpha, Fibroblast activation protein alpha; FGFR2b, Fibroblast growth factor receptor 2b; FOLR1, Folate receptor alpha; GPC3, Glypican-3; HER2, Human epidermal growth factor receptor 2; HER3, Human epidermal growth factor receptor 3; MUC16, Mucin-16; PARP, Poly ADP-ribose polymerase; PD-L1, Programmed death-ligand 1; PSMA, Prostate-specific membrane antigen; ROR1, Receptor tyrosine kinase like orphan receptor 1; ROR2, Receptor tyrosine kinase-like orphan receptor 2; TF, Tissue factor; and TROP2, Trophoblast cell surface antigen 2.

ADC	Target	Payload	Condition/Disease	Other Observations	Clinical Trial Phase
AGX101	TM4SF1	Microtubule inhibitor	Advanced solid tumors	Pancreatic adenocarcinoma, triple-negative breast cancer, gastrointestinal cancers can be therapeutic indications	I
ALX2004	EGFR	Topoisomerase I inhibitor	Advanced or metastatic solid tumors	Non-small cell lung cancer, colorectal cancer, and head and neck squamous cell carcinoma are the main therapeutic indications	I
ARX517	PSMA	Microtubule inhibitor	Metastatic castration-resistant prostate cancer	High serum stability with significant antitumor activity	I/II
AZD0901	CLDN18.2	Microtubule inhibitor	Advanced solid tumors	Advanced gastric or gastroesophageal junction adenocarcinoma can be therapeutic indications	II
AZD4512	CD22	Topoisomerase I inhibitor	Acute lymphoblastic leukemia	Its key advantages include high efficacy, a unique mechanism to overcome resistance, and a well-tolerated safety profile	I/II
BAT8008	TROP2	Topoisomerase I inhibitor	Advanced solid tumors	It includes breast cancer as a therapeutic indication	I
BAY 3547926	GPC3	Radioactive isotope	Advanced hepatocellular carcinoma	The radioactive isotope emits high-energy alpha particles to induce lethal DNA double-strand breaks in tumor cells	I
BC3195	CDH3	Microtubule inhibitor	Advanced or metastatic solid tumors	Non-small cell lung cancer, breast cancer, and esophageal cancer can be therapeutic indications	I
BG-C137	FGFR2b	Topoisomerase I inhibitor	Advanced solid tumors	Gastric and breast cancer can be therapeutic indications	I
BNT329	CA19-9	Topoisomerase I inhibitor	Advanced solid tumors	The CA19-9 is present in cancers of the pancreas, bladder, and ovary	I/II
CAB-ROR2-ADC	ROR2	Microtubule inhibitor	Advanced solid tumors	Lung, breast, and head and neck cancers can be therapeutic indications	II
CRB-701	Nectin-4	Microtubule inhibitor	Advanced solid tumors	Improved stability and reduced payload release in plasma	I/II
DS-7300a	B7-H3 (also known as CD276)	Topoisomerase I inhibitor	Advanced esophageal cancer	Advanced-stage small cell lung cancer, non-small cell lung cancer, and metastatic castration-resistant prostate cancer studies are underway	II
FOR46 (also known as FG-3246)	CD46	Microtubule inhibitor	Metastatic castration-resistant prostate cancer	Beyond direct cytotoxicity, it has been shown to induce immune-priming effects, such as increasing effector CD8+ T cells	II
GSK5733584 (also known as HS-20089)	B7-H4	Topoisomerase I inhibitor	Endometrial cancer	Ovarian and breast cancers can be other therapeutic indications	III
GSK5764227 (also known as HS-20093)	B7-H3	Topoisomerase I inhibitor	Relapsed small cell lung cancer	Relapsed/refractory osteosarcoma can be other therapeutic indication	III
HLX43	PD-L1	Topoisomerase I inhibitor	Advanced solid tumors	Non-small cell lung cancer, cervical cancer, and esophageal squamous cell carcinoma can be therapeutic indications	I
HMBD-501	HER3	Topoisomerase I inhibitor	Advanced-stage, relapsed and/or refractory HER3-expressing solid tumors	It has a stable conjugation profile with increased hydrophilicity	I/II
HS-20110	CDH17	Topoisomerase I inhibitor	Advanced colorectal cancer	Can be used in other solid tumors	I/II
HWK-016-101	MUC16	Topoisomerase I inhibitor	Advanced or metastatic solid tumors	Ovarian and endometrial cancer are the main therapeutic indications	I
IMGN632	CD123	DNA alkylating agent	Acute myeloid leukemia and other CD123-positive hematologic malignancies	Its advantages stem from its unique payload, high selectivity for cancer cells over normal cells, and potent synergistic effects in combination treatments	I/II
JS212	EGFR/HER3	Topoisomerase I inhibitor	Metastatic colorectal cancer	Bispecific ADC	II
LY4170156	FOLR1	Topoisomerase I inhibitor	Ovarian cancer	In addition, it is being studied for peritoneal and fallopian tube cancers	III
M3554	GD2	Topoisomerase I inhibitor	Advanced solid tumors	Neuroblastoma, sarcoma, and glioma can be therapeutic indications	I
M9140	CEACAM5	Topoisomerase I inhibitor	Colorectal cancer	Absence of specific toxicities, high stability, and targeted release	I
MHB088C	B7-H3	Topoisomerase I inhibitor	Advanced solid tumors	It is designed to have superior potency compared to other B7-H3 ADCs, with high internalization rates and high stability	I/II
MRG004A	TF	Microtubule inhibitor	Metastatic or unresectable solid tumors	Cervical cancer, pancreatic cancer, and triple-negative breast cancer can be therapeutic indications	I/II
MRG006A	GPC3	Topoisomerase I inhibitor	Advanced solid tumors	Hepatocellular carcinoma can be a therapeutic indication	I/II
NN3201	c-Kit	Microtubule inhibitor	Advanced and/or metastatic solid tumors	Therapeutic indications can be gastrointestinal stromal tumors and small cell lung cancer	I
OBI-902	TROP2	Topoisomerase I inhibitor	Advanced solid tumors	Orphan drug status is awarded to cholangiocarcinoma	I/II
OBI-992	TROP2	Topoisomerase I inhibitor	Advanced solid tumors	High stability, bystander effect, and synergy (e.g., combined with PARP inhibitors)	I/II
OBT076 (also known as MEN1309)	CD205	Microtubule inhibitor	Recurrent and/or metastatic CD205+ solid tumors	CD205-positive malignancies include pancreatic, bladder, triple-negative breast cancer, and non-Hodgkin lymphoma	I
OMTX705	FAP-alpha	Microtubule inhibitor	Advanced/metastatic pancreatic adenocarcinoma	A strategy for cancer treatment, including tumors resistant to immunotherapy	I
QLS5132	CLDN6	Topoisomerase I inhibitor	Advanced solid tumors	Ovarian and non-small cell lung cancer can be therapeutic indications	I
REGN5093-M114	c-Met	Microtubule inhibitor	Advanced non-small cell lung cancer	Biparatopic ADC, acting through binding to two different and non-overlapping epitopes on the MET receptor	I/II
SAR3419	CD19	Microtubule inhibitor	B-cell malignancies	Primarily studied for B-cell non-Hodgkin lymphoma and acute lymphoblastic leukemia	I/II
SHR-A1811	HER2	Topoisomerase I inhibitor	Locally advanced/metastatic HER2 positive breast cancer	In combination with pyrotinib	II
SKB315	CLDN18.2	Topoisomerase I inhibitor	Advanced solid tumors	Specific use in gastric/gastroesophageal junction cancer	I
STI-6129	CD38	Microtubule inhibitor	Relapsed/refractory multiple myeloma	Amyloid light-chain amyloidosis can be a therapeutic indication	I/II
SYS6010 (also known as CPO301)	EGFR	Topoisomerase I inhibitor	Advanced or metastatic esophageal squamous cell carcinoma	Non-small cell lung cancer can be other therapeutic indication	II/III
TORL-4–500	DLK1	Microtubule inhibitor	Advanced or metastatic solid tumors	Adrenocortical carcinoma can be a therapeutic option	I
TQB2101	ROR1	Topoisomerase I inhibitor	Advanced hematologic malignancies	Advanced solid tumors are a potential therapeutic option	I
TQB2102	HER2	Topoisomerase I inhibitor	Recurrent/metastatic advanced gynecological tumors	Advanced or metastatic non-small cell lung cancer with HER2 gene abnormality can be other therapeutic indication	II
TQB6411	EGFR/c-Met	Topoisomerase I inhibitor	Advanced malignant tumors, including non-small cell lung cancer	Bispecific ADC	I
ZL-1310	DLL3	Topoisomerase I inhibitor	Relapsed small cell lung cancer	Promising antitumor activity, including in brain metastases	III
ZW251	GPC3	Topoisomerase I inhibitor	Advanced solid tumors	Hepatocellular carcinoma can be a therapeutic indication	I

## Data Availability

No new data were created or analyzed in this study. Data sharing is not applicable to this article.

## Abbreviations

The following abbreviations are used in this manuscript:

ADCC	Antibody-dependent cell-mediated cytotoxicity
ADCP	Antibody-dependent cellular phagocytosis
ADCs	Antibody–drug conjugates
ATP	Adenosine triphosphate
CDC	Complement-dependent cytotoxicity
CLDN18.2	Claudin 18.2
DAR	Drug-to-antibody ratios
DNA	Deoxyribonucleic acid
Fab	Fragment antigen-binding
Fc	Fragment crystallizable
HER2	Human epidermal growth factor receptor 2
HER3	Human epidermal growth factor receptor 3
mAbs	Monoclonal antibodies
mRNA	Messenger ribonucleic acid
PROTACs	Proteolysis targeting chimeras
RNA	Ribonucleic acid
scFv	Single-chain variable fragment
STING	Stimulator of interferon genes
TAA	Tumor-associated antigen
TLRs	Toll-like receptors
TROP2	Trophoblast cell surface antigen 2
TSA	Tumor-specific antigen
